# Diagnosing and managing sleep apnea in patients with chronic cerebrovascular disease: a randomized trial of a home-based strategy

**DOI:** 10.1007/s11325-017-1494-5

**Published:** 2017-04-06

**Authors:** Dawn M. Bravata, Vincent McClain, Charles Austin, Jared Ferguson, Nicholas Burrus, Edward J. Miech, Marianne S. Matthias, Neale Chumbler, Susan Ofner, Brian Foresman, Jason Sico, Carlos A. Vaz Fragoso, Linda S. Williams, Rajiv Agarwal, John Concato, H. Klar Yaggi

**Affiliations:** 1 VA HSR&D Center of Excellence on Implementing Evidence-Based Practice (CIEBP), Richard L. Roudebush VA Medical Center, HSR&D Mail Code 11H, 1481 West 10th Street, Indianapolis, IN 46202 USA; 2 VA Health Services Research and Development (HSR&D) Stroke Quality Enhancement Research Initiative (QUERI), Indianapolis, IN USA; 3 Department of Internal Medicine, Indiana University School of Medicine, Indianapolis, IN USA; 4 Department of Neurology, Indiana University School of Medicine, Indianapolis, IN USA; 5 Regenstrief Institute, Indianapolis, IN USA; 6 Department of Emergency Medicine, Indiana University School of Medicine, Indianapolis, IN USA; 7 Department of Communication Studies, Indiana University-Purdue University at Indianapolis (IUPUI), Indianapolis, IN USA; 8 Health Policy and Management, University of Georgia, Athens, GA USA; 9 Department of Biostatistics, Indiana University School of Medicine, IUPUI, Indianapolis, IN USA; 10 Internal Medicine Service, Richard L. Roudebush VA Medical Center, Indianapolis, IN USA; 11 VA Connecticut Healthcare System, West Haven, CT USA; 12 Department of Internal Medicine, Yale University School of Medicine, New Haven, CT USA; 13 Department of Neurology, Yale University School of Medicine, New Haven, CT USA; 14 Clinical Epidemiology Research Center, VA Connecticut Healthcare System, West Haven, CT USA

**Keywords:** Ischemic stroke, Transient ischemic attack, Sleep apnea diagnosis, Sleep apnea treatment

## Abstract

**Background:**

Obstructive sleep apnea is common and associated with poor outcomes after stroke or transient ischemic attack (TIA). We sought to determine whether the intervention strategy improved sleep apnea detection, obstructive sleep apnea (OSA) treatment, and hypertension control among patients with chronic cerebrovascular disease and hypertension.

**Methods:**

In this randomized controlled strategy trial intervention, patients received unattended polysomnography at baseline, and patients with OSA (apnea-hypopnea index ≥5 events/h) received auto-titrating continuous positive airway pressure (CPAP) for up to 1 year. Control patients received usual care and unattended polysomnography at the end of the study, to identify undiagnosed OSA. Both groups received 24-h blood pressure assessments at baseline and end of the study. “Excellent” CPAP adherence was defined as cumulative use of ≥4 h/night for ≥70% of the nights.

**Results:**

Among 225 randomized patients (115 control; 110 intervention), 61.9% (120/194) had sleep apnea. The strategy successfully diagnosed sleep apnea with 97.1% (102/105) valid studies; 90.6% (48/53, 95% CI 82.7–98.4%) of sleep apnea was undiagnosed among control patients. The intervention improved long-term excellent CPAP use: 38.6% (22/57) intervention versus 0% (0/2) control (*p* < 0.0001). The intervention did not improve hypertension control in this population with well-controlled baseline blood pressure: intervention, 132.7 mmHg (±standard deviation, 14.1) versus control, 133.8 mmHg (±14.0) (adjusted difference, −1.1 mmHg, 95% CI (−4.2, 2.0)), *p* = 0.48).

**Conclusions:**

Patients with cerebrovascular disease and hypertension have a high prevalence of OSA. The use of portable polysomnography, and auto-titrating CPAP in the patients’ homes, improved both the diagnosis and the treatment for sleep apnea compared with usual care but did not lower blood pressure.

## Introduction

Obstructive sleep apnea is found in at least half of individuals who have experienced an acute stroke or transient ischemic attack (TIA) [1–9]. Sleep apnea has been associated with a variety of adverse disease states and outcomes including hypertension, diabetes, ischemic stroke and transient ischemic attacks (TIA), incident coronary events and cardiovascular mortality, and all-cause mortality [3, 10–14]. Estimates suggest, however, that as many as 70–80% of the patients with sleep apnea are neither diagnosed nor treated [15]. The barriers to diagnosing and treating sleep apnea involve patient, provider, and system issues, including access to sleep laboratory-based testing [15].

The American Heart Association/American Stroke Association secondary stroke prevention guidelines recommend that patients with ischemic stroke and TIA receive screening for sleep apnea and treatment because of data suggesting that post-cerebrovascular event patient outcomes are improved with treatment of sleep apnea [16]. However, this guideline recommendation has not been adopted widely. For example, a recent report found that only 6% of the eligible patients received polysomnography in the year after an index cerebrovascular event [17].

The “Diagnosis and Treatment of Sleep Apnea in Cerebrovascular Disease” (*GoToSleep*) study evaluated a strategy to improve the diagnosis and treatment of sleep apnea among veterans with chronic ischemic stroke or TIA who also have hypertension [18]. Specifically, the *GoToSleep* study was designed to overcome some of the barriers that existed within the Veterans Health Administration (VHA) to the timely diagnosis and treatment of sleep apnea (e.g., limited mobility, travel distance) by using home-based ambulatory polysomnography and auto-titrating continuous positive airway pressure (CPAP) to reduce the reliance on laboratory-based sleep studies. The primary objectives of the *GoToSleep* study were to determine whether a diagnostic and therapeutic intervention strategy among veterans with chronic cerebrovascular disease and hypertension could improve: (1) the detection of sleep apnea; (2) the treatment for obstructive sleep apnea; and (3) the control of hypertension.

## Design and methods

The *GoToSleep* study was a randomized controlled strategy trial conducted at two geographically distant Veterans Health Affairs (VHA) medical centers. The methods of this study have been described in detail elsewhere [18]. Briefly, patients with chronic cerebrovascular disease and hypertension were randomly assigned to an intervention group or a usual care control group. The randomization was stratified by medical center, baseline blood pressure, and risk of sleep apnea from the Berlin Questionnaire [19]. This study (NCT00984308) received institutional review board approval; patients provided written informed consent.

### Chronic cerebrovascular disease and hypertension patient definition

Patients were included if they had a history of ischemic stroke or TIA *and* either a diagnosis of hypertension or a blood pressure of ≥140/≥90 mmHg [20]. This study was designed prior to the revision of the American Heart Association/American Stroke Association definition of TIA, and employed the following definitions: an ischemic stroke is a persistent focal neurological deficit of presumed ischemic origin lasting more than 24 h [21] and a TIA is a focal neurological deficit of presumed ischemic origin lasting less than 24 h [22]. Brain imaging was not required for the diagnosis of stroke or TIA. Patients were recruited at least 30 days after their most recent stroke and at any point after their most recent TIA.

### Measurements

Patient interviews and medical record reviews were conducted to obtain information about demographics, comorbidities, medication use, and symptoms. Patients were examined to measure heart rate, neck circumference, height, and weight. The Berlin Questionnaire was used to classify patients according to “high” versus “low” risk for sleep apnea [19]. Daytime sleepiness was assessed using the Epworth Sleepiness Scale (ESS) with a score of ≥10 indicating excessive daytime sleepiness [23, 24]. Neurological functioning was measured with the National Institutes of Health Stroke Scale (NIHSS) [25, 26].

#### Blood pressure and antihypertensive medications

Blood pressure measurements were taken at baseline and at the end of the study with a 24-h ambulatory monitor. Blood pressure data were classified as either sleeping or waking on the basis of actigraphy data or sleep diary information for patients with incomplete actigraphy data. Nocturnal blood pressure dipping patterns were assessed by comparing mean systolic blood pressure during sleep with mean systolic pressure during the wakeful state [27]. Antihypertensive medications were not changed as part of the study but were recorded at the time of the blood pressure measurements. The defined daily dose (DDD) of all antihypertensive medications was calculated using the World Health Organization (WHO) DDD methodology which allows for comparison across antihypertensive regimens [28]. The medication-adjusted mean systolic blood pressure was calculated from the 24-h mean systolic blood pressure measurement and the patient’s DDD as follows: medication-adjusted systolic blood pressure (SBP) = [mean systolic blood pressure in mmHg] + [the patient’s DDD × (8.0 mmHg)] [18].

#### Polysomnography

Intervention patients received full unattended (type 2) polysomnography at baseline (Safiro, Compumedics, Victoria, Australia). Control patients received polysomnography at the end of the 1-year study period. Nearly all of the patients received the polysomnography in their homes. Full unattended polysomnography provided data on electroencephalogram (EEG), electrooculogram, chin electromyogram, thoracic and abdominal displacement, airflow, finger pulse oximeter, electrocardiogram, body position, leg movement, and audio recording of the participant’s snoring. The polysomnographic data were read centrally and scored according to standard criteria [29]. An apnea-hypopnea index (AHI) was calculated as the number of respiratory events per hour of sleep [29–31]. Patients with an AHI ≥5 events/h were diagnosed as having sleep apnea and were offered auto-titrating CPAP [30]. Patients classified as having central sleep apnea or Cheyne-Stokes respiration were continued in the study but were referred for adaptive servoventilation (ASV). Patients with oxygen desaturations by polysomnography received one night of oximetry while using CPAP; patients with persistent desaturations despite auto-titrating CPAP were provided supplemental nocturnal oxygen via the CPAP machine.

### CPAP adherence support

Our approach to improving CPAP adherence consisted of early intensive education and support, followed by regular and ongoing contact with patients. During the in-home visit where the results of the baseline polysomnography were reviewed, staff members provided patients with information about sleep apnea and CPAP. During the first month after delivery of the CPAP machine, the patient was visited each of the first 2 days and then weekly for the first month; thereafter, patients received monthly telephone calls for the remainder of the 1-year study period. An in-home visit was scheduled after 6 months of CPAP use and also at the end of the study. During the in-home visits, staff downloaded pressure, residual AHI, air leak, and compliance data from the CPAP machine and reviewed these data with the patients. Staff members were also available to patients for ad hoc issues related to difficulties with mask fit or other technical problems with the CPAP machines.

### Usual care

Control patients received usual care as directed by their physicians, including care of their vascular risk factors such as hypertension and referral for usual care sleep apnea testing. Control patients in the *GoToSleep* study were neither prohibited from nor specifically encouraged to receive polysomnography as part of their usual care. Control patients received unattended polysomnography at the end of the study period.

### Outcomes

#### Sleep apnea diagnosis

The proportion of intervention and control patients with a diagnosis of obstructive or central sleep apnea was calculated from the unattended polysomnography performed in the intervention group and from any polysomnography that was performed as part of usual care among control patients. The primary outcome related to sleep apnea detection was the prevalence of undiagnosed sleep apnea in the control group which was determined using polysomnography data obtained as part of the research protocol at the end of the study.

#### Obstructive sleep apnea treatment

CPAP adherence was classified as “none” if the patient refused to take receipt of a CPAP machine or did not use the machine at all, “poor” if the nights of usage was ≤10% of total nights available, “some” if the total nights used >10% of total nights available or the cumulative hours of use was <4 h per night times 70% of the total nights available for use, and “excellent” if the total cumulative hours of use was ≥4 h per night times 70% of the total nights available for use [32]. The primary outcome related to obstructive sleep apnea treatment was the proportion of intervention and control patients with obstructive sleep apnea with excellent CPAP adherence.

#### Hypertension control

The primary hypertension outcome was the final mean 24-h SBP, adjusted for baseline mean 24-h SBP. Our secondary hypertension analyses included the final medication-adjusted SBP, the final DDD, and the proportion of patients with a nocturnal decline (“dipping”) in systolic blood pressure of ≥10% [27].

### Sample size

The *GoToSleep* trial was designed to recruit 318 patients to have at least 80% power to detect the following pre-specified differences in the three primary aims: 60% of the intervention patients compared with 10% of the control patients would be diagnosed with sleep apnea; 50% of the intervention patients diagnosed with sleep apnea as part of the study protocol, compared with 15% of the control patients diagnosed with sleep apnea as part of usual care, would use CPAP therapy; and the 1-year mean systolic blood pressure would be 125 mmHg with a standard deviation of 20 mmHg in the intervention group compared with 135 mmHg with a standard deviation of 20 mmHg in the control group. The sample size estimates assumed a 10% loss to follow-up in both the intervention and the usual care arms.

### Analyses

The primary analyses were designed to be intention-to-treat comparisons of the outcomes for patients in the intervention versus control groups. For the analysis of diagnosis rates, all patients were analyzed according to the group to which they had been randomly assigned regardless of subject study completion. Ninety five percent confidence intervals were calculated around the undiagnosed sleep apnea rate obtained from the control group. For the analysis of treatment rates, CPAP adherence was assessed among patients with obstructive sleep apnea. The comparison of final mean 24-h SBP was not restricted to patients with sleep apnea, but rather was conducted across all patients with final ambulatory blood pressure data, regardless of sleep study results. A secondary hypertension control analysis was pre-specified to be conducted among sleep apnea patients in the intervention group with or without excellent CPAP adherence, to examine the effect of CPAP use on antihypertensive outcomes.

Baseline characteristics were compared between intervention and control groups using Fisher’s exact test, chi-squared test, 2-sample *t* test, or Wilcoxon 2-sample tests. Chi-squared tests were used to test for group differences in diagnosis and treatment rates. An analysis of covariance (ANCOVA) model, with terms for site, group, and baseline measure, was used to test for differences in mean final 24-h SBP. Residual plots were examined for possible violation of model assumptions. The final medication-adjusted SBP was analyzed similarly. Final DDD was rank transformed because of skewness and was analyzed similarly, including a squared baseline DDD term. Summary statistics of change in measures are presented but were not modeled or statistically tested, because the analyses of the final measurements included adjustment for baseline measurements. A logistic model with terms for site and group was used to estimate the effect of the intervention on the odds of having nocturnal decline of ≥10%. A cumulative logistic model was used for the ordinal outcome of nocturnal dipping category and modeled the probability of having greater nighttime dipping. The proportional odds assumption was verified. The secondary analyses of hypertension control measures by CPAP adherence used similar models with the term for treatment group replaced by a variable for CPAP adherence (excellent versus not). No corrections for multiple comparisons were made, because we pre-specified a single primary outcome for the three primary aims. SAS® version 9.2 (Cary, NC) was used for all analyses.

## Results

**Fig. 1:**
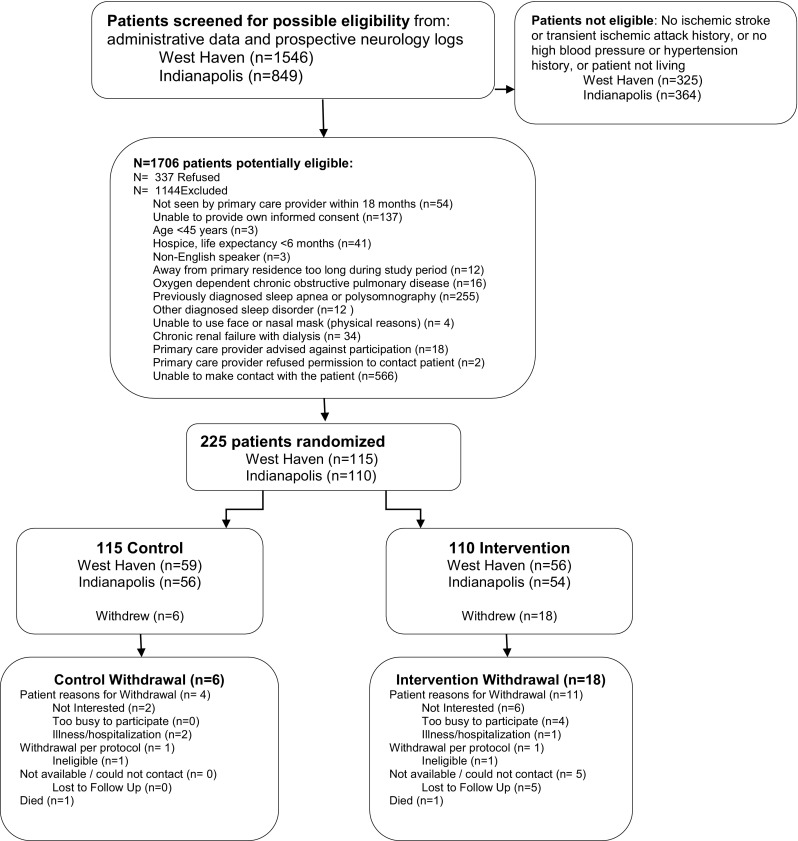
Patient screening, recruitment, and retention flow

The medical records of *N* = 2395 patients were screened for eligibility. Among the *N* = 1706 (71.2%) potentially eligible patients, 337 (19.8%) refused and 1144 (67.1%) met at least one exclusion criterion (Fig. 1). A total of 225 patients were included in the study: *N* = 110 intervention and *N* = 115 control. Overall, 24/225 (10.7%) patients withdrew: 6 (5.2%) from the control group and 18 (16.4%) from the intervention group. The baseline characteristics of the patients are provided in Table 1.

**Table 1:** Baseline characteristics

Characteristic	Intervention (*N* = 110)	Control (*N* = 115)	*p* value
Age (years): (range), median	(45, 91) 70	(50, 90) 70	
Mean ± standard deviation	69.7 ± 10.4	70.2 ± 10.1	0.81
White race: *N* (%)	82 (74.6)	92 (80.0)	0.33
Male sex: *N* (%)	109 (99.1)	110 (95.7)	0.21
Excessive daytime sleepiness (ESS ≥10): *N* (%)	36 (32.4)	28 (24.1)	0.17
Index cerebrovascular event^a^			
Stroke: *N* (%)	75 (68.2)	87 (75.7)	0.21
Transient ischemic attack (TIA): *N* (%)	49 (44.6)	45 (39.1)	0.41
Time from most recent event to enrollment (year): (range) median	(0, 44) 4	(0, 65) 4	
Mean ± standard deviation	6.2 ± 7.4	7.3 ± 9.2	0.45
Comorbidity: *N* (%)			
Hypertension	110 (100.0)	115 (100.0)	
On any antihypertensive medication	104 (94.6)	111 (96.5)	0.53
Hyperlipidemia	88 (80.0)	96 (83.5)	0.50
Diabetes mellitus	51 (46.4)	40 (34.8)	0.08
Chronic pain	42 (38.2)	31 (27.0)	0.07
Depression	39 (35.5)	37 (32.2)	0.60
Current tobacco smoking: *N* (%)	32 (29.1)	32 (27.8)	0.83
Chronic obstructive pulmonary disorder (COPD)	26 (23.6)	21 (18.3)	0.32
Myocardial infarction	25 (22.7)	30 (26.1)	0.56
Anxiety	23 (20.9)	13 (11.3)	0.05
Peripheral vascular disease	19 (17.3)	26 (22.6)	0.32
Atrial fibrillation	11 (10.0)	19 (16.5)	0.15
Chronic kidney disease	10 (9.1)	11 (9.6)	0.90
Dementia	10 (9.1)	6 (5.2)	0.26
Congestive heart failure	9 (8.2)	14 (12.2)	0.32
Charlson comorbidity score: mean ± standard deviation	2.6 ± 2.2	2.3 ± 2.2	0.25
Measurements			
Neck circumference (inches): mean ± standard deviation	16.4 ± 1.5	16.3 ± 1.3	0.87
Waist circumference (inches): mean ± standard deviation	41.3 ± 5.2	42.2 ± 5.1	0.19
Weight (pounds): mean ± standard deviation	188.6 ± 34.3	193.4 ± 34.7	0.19
Body mass index (kg/m^2^): mean ± standard deviation	38.5 ± 12.9	39.6 ± 12.9	0.45
NIH Stroke Scale: (range) median	(0, 12), 1.0	(0,19), 1.0	
Mean ± standard deviation	2.2 ± 2.7	2.0 ± 3.1	0.51
Systolic blood pressure (mmHg): (range) median	(86, 184) 132	(94, 190) 134	
Mean ± standard deviation	132.1 ± 19.3	133.8 ± 17.3	0.43
Diastolic blood pressure (mmHg): (range) median	(36, 100) 72	(50, 102) 72	
Mean ± standard deviation	71.6 ± 11.9	72.5 ± 11.3	0.72
24-h mean systolic blood pressure (mmHg): range, median	(97.2, 176.5) 134.4	(98.7, 178.5) 136.8	
Mean ± standard deviation	134.2 ± 14.1	136.1 ± 13.3	0.32
24-h mean diastolic blood pressure (mmHg): range, median	(51.7, 97.5) 72.0	(51.1, 116.8) 72.2	
Mean ± standard deviation	72.3 ± 10.1	73.8 ± 11.4	0.48
Antihypertensive medication defined daily dose (DDD): (range), median	(0.0, 9.5) 2.7	(0.0, 9.7) 2.7	
Mean ± standard deviation	2.9 ± 2.3	3.1 ± 2.3	0.43
Medication-adjusted SBP (mmHg): range, median	(116.3, 226.8) 155.0	(100.7, 232.0) 157.4	
Mean ± standard deviation	157.4 ± 25.0	161.6 ± 24.3	0.17
Nocturnal blood pressure dipping patterns: *N* (%)			
Extreme: ≥20% fall in SBP at night/sleep	3 (3.1)	5 (4.9)	0.60
Normal: ≥10 but <20% fall in SBP at night/sleep	21 (21.7)	15 (14.6)	
None: <10 to ≤0% fall at night/sleep	43 (44.3)	50 (48.5)	
Reverse: SBP, sleep >wakefulness	30 (30.9)	33 (32.0)	

*ESS* Epworth Sleepiness Scale, *SBP* systolic blood pressure

^a^All patients in this cohort had a history of a cerebrovascular event and hypertension

### Sleep apnea diagnosis rate

**Fig. 2:**
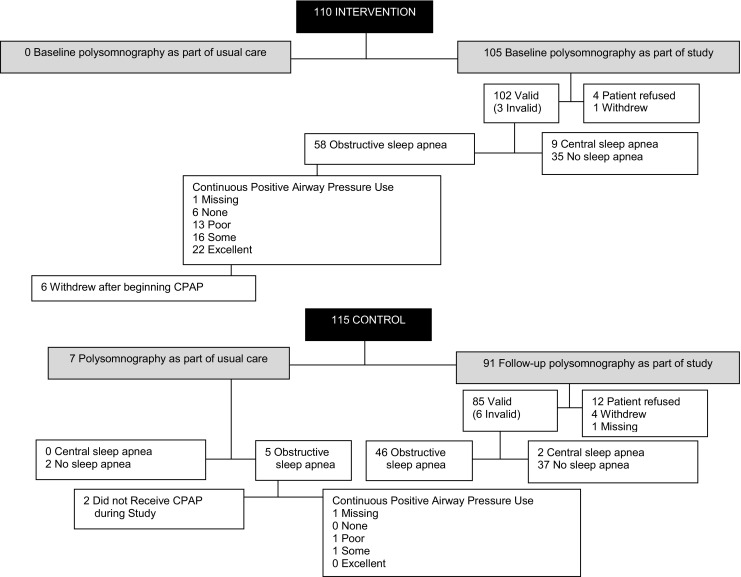
Receipt of polysomnography and continuous positive airway pressure

Polysomnography was conducted at the beginning of the study on 105 of the 110 intervention patients (Fig. 2); 102/105 (97.1%) of these studies were valid (three studies had insufficient high-quality data to be scored). Among the 102 valid studies, 58 (56.9%) had obstructive sleep apnea, 9 (8.8%) had central sleep apnea, and 35 (34.3%) had no sleep apnea. Polysomnography was completed as part of usual care in 7 of the 115 control patients (7/115, 6.1%); 5/7 (71.4%) had obstructive sleep apnea, none had central sleep apnea, and 2/7 (28.6%) had no sleep apnea. Polysomnography was performed at the end of the study, as part of the research protocol, on 91 control patients; 85/91 (93.4%) were valid studies. Among the 85 valid studies obtained as part of the study protocol among control patients, 46 (54.1%) had obstructive sleep apnea, 2 (2.4%) had central sleep apnea, and 37 (43.5%) had no sleep apnea. A total of 53 patients had sleep apnea in the control group: 51 with obstructive sleep apnea (5 diagnosed as part of usual care and 46 diagnosed as part of the study protocol) and 2 with central sleep apnea (all diagnosed as part of the study protocol). The prevalence of undiagnosed sleep apnea (from the control group) was 90.6% (48/53; 95% CI, 82.7 to 98.4%) (Tables 2 and 3).

**Table 2:** Polysomnographic data

Characteristic	Overall	Intervention	Control
Number of valid sleep studies	(*n* = 194)	(*n* = 102)	(*n* = 92)^a^
Apnea-hypopnea index (AHI, events/h): (range) median	(0, 87.2) 7.5	(0, 87.2) 7.6	(0, 64.9) 7.3
Mean ± standard deviation	14.3 ± 16.1	15.0 ± 16.6	13.6 ± 15.7
Central apnea index: mean ± standard deviation	2.2 ± 6.7	2.9 ± 8.4	1.3 ± 3.5
Polysomnography results: *N* (%)			
Obstructive sleep apnea	109 (56.2)	58 (56.9)	51 (55.4)
AHI: mean ± standard deviation	21.2 ± 15.6	20.0 ± 15.2	22.6 ± 16.1
Central sleep apnea	11 (5.6)	9 (8.8)	2 (2.2)
No sleep apnea	74 (38.1)	35 (34.3)	39 (42.4)

^a^The valid sleep studies in the control group include the 85 obtained as part of the study and 7 obtained as part of routine clinical care

**Table 3:** Detection of sleep apnea rates

Population	Intervention (*N* = 110)	Control (*N* = 115)	*p* value	Difference	95% CI
Sleep apnea identified among all patients	67/110 (60.9%)	5/115 (4.4%)	<0.0001	56.6%	(46.7, 66.4)
Sleep apnea identified among patients with sleep apnea	67/67 (100.0%)	5/53 (9.4%)	<0.0001	90.6%	(82.7, 98.4)

Sleep apnea refers to either obstructive or central sleep apnea

Overall (including both intervention and control patients), 61.9% (120/194) of the patients had obstructive or central sleep apnea. The ESS was ≥10 in 40/119 (33.6%) with sleep apnea and 23/75 (30.7%) without sleep apnea (*p* = 0.67). The Berlin score was classified as high risk in 85/119 (71.4%) with sleep apnea and 49/75 (65.3%) without sleep apnea (*p* = 0.37).

### Obstructive sleep apnea treatment rate

Among the five control patients who were diagnosed with obstructive sleep apnea as part of usual care (see Fig. 2), two patients did not receive CPAP during the study and one patient had missing CPAP use data. Among the two control patients with known CPAP use data, 0 (0%) had excellent CPAP adherence; the mean number of hours used per night was 0.1 h (standard deviation 0.2); and the mean proportion of nights with any CPAP use was 0.07 (standard deviation 0.08). In contrast, among the 58 intervention patients who were diagnosed with obstructive sleep apnea during the study, 1 patient had missing CPAP use data, 22 (38.6%) had excellent CPAP adherence, the mean number of hours used per night was 2.5 h (standard deviation 2.8), and the proportion of nights with any CPAP use was 0.45 (standard deviation 0.40). The intervention patients were much more likely to have excellent CPAP adherence than the control patients: 38.6 (22/57) versus 0% (0/2), *p* < 0.0001.

**Fig. 3:**
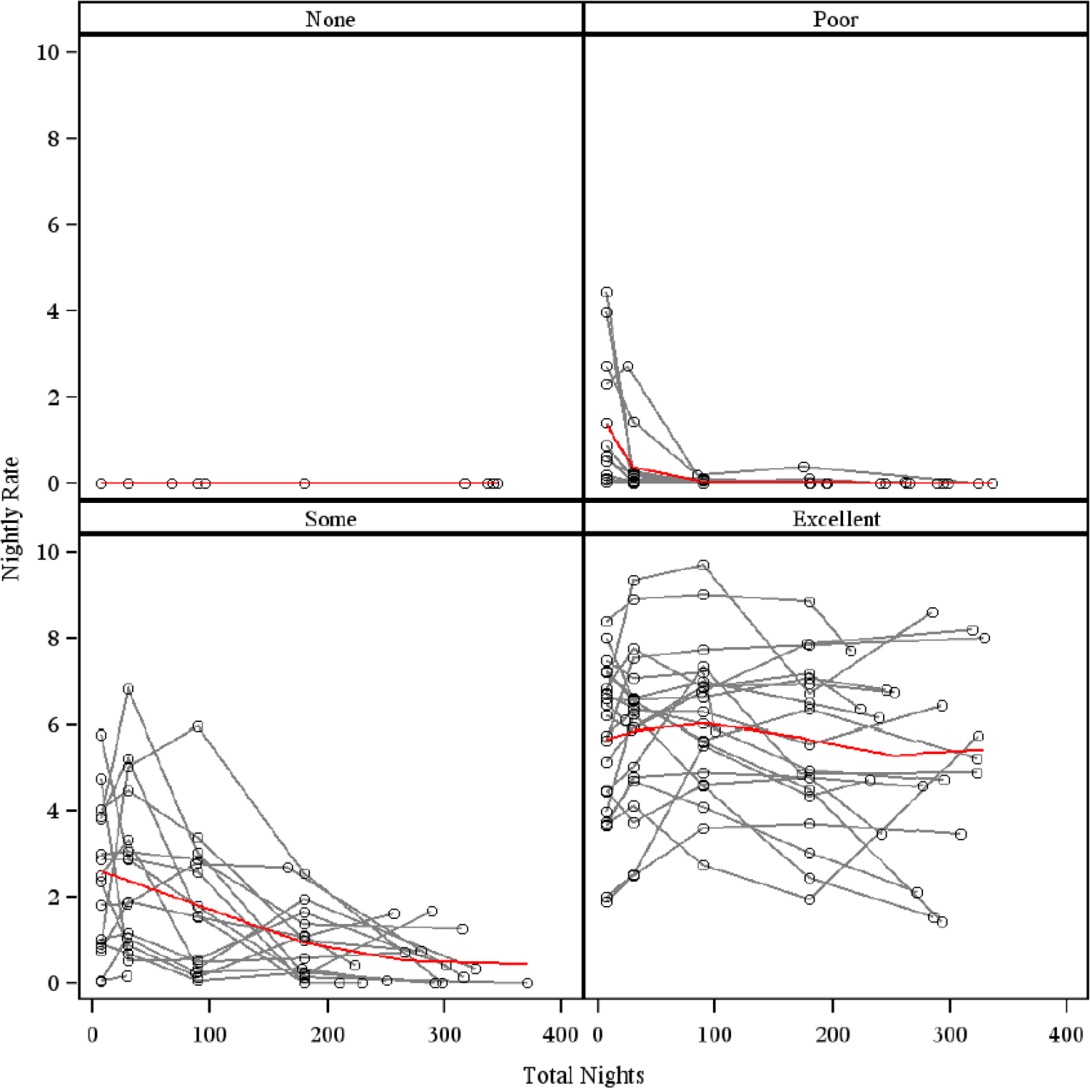
Patterns of continuous positive airway pressure over a 1-year period

Figure 3 provides a graphical illustration of CPAP adherence over time with hours per night used on the *y*-axis and nights in the study on the *x*-axis. These data suggest that CPAP use varied considerably over time and that there was no consistent point in time after which CPAP use stabilized.

### Hypertension control

The blood pressure was relatively well controlled at baseline in this population of patients (Table 1). The baseline mean 24-h systolic blood pressure was 134.2 mmHg (±14.1) for intervention patients and 136.1 mmHg (±13.3) for control patients (*p* = 0.32). The overwhelming majority of patients (94.6% of the intervention patients and 96.5% of the control patients, *p* = 0.53) were taking at least one antihypertensive medication. The intervention strategy did not significantly improve blood pressure control; the final mean 24-h systolic blood pressure was nearly identical in the two groups: intervention, 132.7 mmHg (± standard deviation of 14.1); control, 133.8 mmHg (±14.0; *p* = 0.48; Table 4). When restricting the analysis to intervention patients with sleep apnea, no difference in the change in mean 24-h systolic blood pressure was identified for patients with excellent (−1.1 mmHg) versus not excellent (−1.1 mmHg) CPAP adherence (Table 5). Both final and change in medication-adjusted 24-h SBP favored the excellent use group, but the difference between mean final medication adjusted SBP was not statistically significant (excellent, 149.4 mmHg; not excellent 166.9 mmHg; *p* = 0.40).

**Table 4:** Hypertension control: intention to treat analysis

Blood pressure outcomes	Intervention (*N* = 110)	Control (*N* = 115)	Adjusted final means (standard error)		Adjusted mean difference (95% CI) intervention versus control	*p* value
			Intervention	Control		
Final mean 24-h SBP (mmHg): (range), median	(102.4, 173.7) 132.0	(107.9, 174.1) 132.9	132.8 (1.2)	133.9 (1.1)	−1.1 (−4.2, 2.0)	0.48
Mean ± standard deviation	132.7 ± 14.1	133.8 ± 14.0				
Change in mean 24-h SBP (mmHg): (range), median	(−44.4, 29.4) −0.5	(−42.9, 26.3) −0.1				
Mean ± standard deviation	−2.4 ± 12.5	−1.6 ± 11.3				
Final DDD: (range), median	(0.0,10.1) 2.5	(0.0,11.5) 2.7	3.1 (0.1)	3.1 (0.1)	0.0 (−0.4, 0.4)	0.51
Mean ± standard deviation	3.0 ± 2.5	3.2 ± 2.2				
Change in DDD: (range), median	(−3.0, 4.8) 0.0	(−5.0, 5.3) 0.0				
Mean ± standard deviation	0.1 ± 1.2	0.0 ± 1.5				
Final medication-adjusted SBP (mmHg): (range), median	(112.8, 240.1) 154.1	(110.0, 248.5) 156.8	158.4 (1.7)	158.7 (1.6)	−0.2 (−4.8, 4.4)	0.93
Mean ± standard deviation	157.3 ± 28.2	158.8 ± 25.5				
Change in medication-adjusted SBP (mmHg): (range), median	(−50.6,39.8) −0.2	(−42.9, 45.0) 0.5				
Mean ± standard deviation	−1.3 ± 16.7	−1.3 ± 15.8				
Final nocturnal blood pressure dipping patterns: *n* (%)					Odds ratio (95% CI)	
Extreme: ≥20% fall in SBP at night/sleep	2 (1.8)	2 (1.7)			0.96 (0.55, 1.71)	0.90
Normal: ≥10 but <20% fall in SBP at night/sleep	16 (14.6)	17 (14.8)				
None: <10 to ≤0% fall at night/sleep	40 (36.4)	49 (42.6)				
Reverse: SBP at night/sleep greater than daytime/awake	22 (20.0)	23 (20.0)				

Modeling the probability of having greater nighttime dipping

**Table 5:** Hypertension control: among sleep apnea patients

Blood pressure outcomes	Continuous positive airway pressure adherence		Adjusted final means (standard error)		Adjusted mean difference (95% CI) excellent versus not	*p* value
	Not excellent (*N* = 35)	Excellent (*N* = 22)	Not excellent	Excellent		
Final mean 24-h SBP (mmHg): (range), median	(109.5, 173.7) 134.4	(102.4, 161.7) 128.8	132.9 (2.4)	131.6 (2.5)	−1.3 (−8.1, 5.6)	0.71
Mean ± standard deviation	135.2 ± 13.5	129.5 ± 14.8				
Change in mean 24-h SBP (mmHg): (range), median	(−23.9, 29.4) 1.2	(−26.6, 18.0) −0.4				
Mean ± standard deviation	−1.1 ± 13.1	−1.1 ± 11.2				
Final DDD: (range), median	(0.2, 10.1) 3.0	(0.0, 9.3) 2.5	3.1 (0.2)	2.9 (0.3)	−0.3 (−0.9, 0.4)	0.41
Mean ± standard deviation	3.4 ± 2.5	2.5 ± 2.2				
Change in DDD: (range), median	(−3.0, 3.1) 0.0	(−3.0, 2.0) 0.0				
Mean ± standard deviation	0.0 ± 1.3	−0.3 ± 1.1				
Final medication-adjusted SBP (mmHg): (range), median	(120.0, 240.1) 164.0	(112.8, 236.1) 146.6	161.3 (3.5)	156.9 (3.7)	−4.4 (−14.7. 6.0)	0.40
Mean ± standard deviation	166.9 ± 28.4	149.4 ± 25.6				
Change in medication-adjusted SBP (mmHg): (range), median	(−37.4, 39.8) 2.1	(−50.6, 20.5) −3.2				
Mean ± standard deviation	0.6 ± 16.9	−3.3 ± 15.5				
					Odds ratio (95% CI)	
Proportion with final nocturnal decline >10%: *N* (%)	3 (8.6%)	5 (22.7%)			1.4 (0.6, 3.0)	0.44

## Discussion

These results demonstrate that sleep apnea is very common among patients with chronic cerebrovascular disease and hypertension; the observed prevalence of 120/194 (62%) is similar to rates observed in studies that have primarily focused on acute and subacute cerebrovascular disease populations [1–9]. These results also demonstrate that a strategy of diagnosing and treating sleep apnea in the homes of patients with chronic cerebrovascular disease and hypertension can lead to substantial improvements in the detection of sleep apnea and in treatment with CPAP.

Usual care identified less than one in ten patients who actually had sleep apnea, a rate that leaves considerable room for improvement. Moreover, the sleep apnea that was detected in this study population included some patients with very high AHIs indicative of severe disease; therefore, it is incorrect to assume that only mild sleep apnea remained undetected by usual care. Only 64/225 (28%) participants in this study had excessive daytime sleepiness according to the Epworth Sleepiness Scale, indicating that “typical” patient characteristics that might alert primary care providers to the need for performing polysomnography were uncommon. Specifically, neither the Epworth Sleepiness Scale nor the Berlin Questionnaire was useful in identifying patients with sleep apnea in this study. It is unknown whether aging or cerebrovascular disease may contribute to a reduced perception of drowsiness. In the absence of validated and reliable methods of screening for polysomnography in the post-stroke and post-TIA populations [33, 34], it is reasonable to use polysomnography to identify sleep apnea given the high underlying prevalence of sleep apnea in this population and the known consequences of untreated sleep apnea.

Because testing for sleep apnea was so uncommon in the usual care group in this study, the number of patients who were eligible for treatment as part of usual care was very small; this limited our assessment of CPAP adherence in usual care versus the intervention groups. None of the usual care patients used CPAP for the recommended ≥4 h per night for ≥70% of the nights, whereas 38.6% (22/57) of the intervention patients achieved this degree of adherence. The two VHA medical centers differed in their approach to usual care. One provided CPAP mask fitting and education via a contract with a home health agency, and the other provided these services via an on-site durable medical equipment (DME) CPAP clinic. Both sites provided a visit with a trained sleep staff member after 30 days, and as-needed support related to mask fit or equipment functioning. In general, usual care at these two sites is similar to sleep care received in the non-VHA setting. The intervention patients, in contrast, had both more frequent contact with staff and the content of the contact was different from usual care. In particular, the staff worked with patients to identify salient motivators for using CPAP (e.g., having more energy to play with grandchildren) and salient barriers inhibiting CPAP use (e.g., nasal stuffiness). As the patients began to use the CPAP, their salient positives and negatives might have changed, but the staff consistently sought to identify methods to overcome those barriers and remind patients of their individual reasons for trying the CPAP. In this way, the content of the visits was not only focused on technical issues related to mask fit, leak, and humidification but also on motivators to try CPAP and to persist over time. In this study, CPAP adherence was not associated with physical impairments (data not shown). Even patients with hemiparesis and blindness were able to achieve excellent CPAP adherence with adequate support from study staff and from caregivers.

This study did not identify a difference in blood pressure management for intervention versus control patients. Three key reasons likely contributed to this negative finding. First, the sample size of this trial was designed to detect a 10-mmHg difference in mean 24-h systolic blood pressure and this study did not achieve the anticipated sample size (*N* = 225 versus *n* = 318). Our inability to achieve the targeted sample size was principally due to delays in hiring and training needed staff within the VHA human resource constraints and because patients who were included in the study lived further away from the medical center than in our pilot work; hence, the resources needed (e.g., staff time, mileage reimbursement) to provide in-home polysomnography and CPAP support were greater than expected. Moreover, several meta-analyses have been published since the time of designing this trial which suggest that the actual difference in systolic blood pressure that can be expected with CPAP ranges from −1.4 to −7.2 mmHg [35–38], with most of the estimates closer to the 1.4 mmHg end of the range. A sample size of 167 patients with sleep apnea in each group would have been required to demonstrate a 5-mmHg difference in mean 24-h systolic blood pressure among excellent versus not excellent CPAP users. A much larger sample size would have been required to identify a statistically significant relationship between intervention versus control group patients with a systolic blood pressure change in the 1.5- to 2.0-mmHg range [35–38]. Second, this population of veterans with hypertension was treated in the VHA primary care setting, and the overwhelming majority was taking antihypertensive medications and their blood pressure was generally very well controlled. The VHA system has placed an emphasis on hypertension care quality, and blood pressure management is an important performance measure within the VHA primary care system nationwide. A recent study of the effect of CPAP on blood pressure among patients with resistant hypertension demonstrated a 3.1-mmHg difference in 24-h systolic blood pressure, but in that study, baseline blood pressures were approximately 10 mmHg higher than in our population (24-h systolic blood pressure of 144.2 mmHg) [39]. Third, we appreciate that hypertension may be attributed to many causes. Because we did not seek to identify etiologies of hypertension, we were not able to identify specific patients in whom sleep apnea may have been the primary etiology of the hypertension.

The primary limitation of the *GoToSleep* trial is its focus on the veteran population, which may limit generalizability to women as there were relatively few female patients in this study. The patients in this trial all had a history of cerebrovascular disease and hypertension, so these results should not be generalized to the acute cerebrovascular event setting—where sleep apnea may be more prevalent and where hemodynamic fluctuations may be more prominent [1–9, 13, 40, 41]. Another potential limitation is that although the NIHSS in this population ranged from 0 to 19, the mean values of approximately 2 suggest that the population had relatively mild stroke severity. Therefore, these results may not be generalizable to a population of chronic cerebrovascular disease patients with more prominent neurological impairments.

Several randomized clinical trials and observational cohort studies have examined the use of CPAP in the cerebrovascular disease population for the purpose of improving post-stroke or post-TIA outcomes; in general, these studies have focused on patients in the acute or subacute event setting. The randomized controlled trials have all been relatively small, with sample sizes insufficient to identify changes in outcomes associated with treatment [40, 42, 43]. The largest of the randomized trials was conducted by Parra et al. [44] and followed (*N* = 126) acute stroke patients with sleep apnea over a 2-year period. The stroke rate was similar in both groups: 3/57 (5.3%) fixed pressure CPAP and 3/69 (4.3%) control (*p* = 1.0); however, the mean time from stroke onset to the first cardiovascular event was longer in the CPAP group: 15 months in the CPAP patients versus 8 months in the control patients (*p* = 0.044). Two randomized controlled trials evaluated the use of CPAP in subacute stroke patients and reported mixed results, finding improvements in several outcome domains for the CPAP group (e.g., depression) versus usual care, and also no statistically significant differences in several outcomes [45, 46].

In conclusion, this study identified both a high prevalence (62%) of sleep apnea in a chronic post-stroke and post-TIA population with hypertension and a very low sleep apnea detection rate (9%) for patients in the usual care group. This study further demonstrated that in-home diagnostic and treatment strategies can be effectively deployed to supplement sleep medicine services that are provided in a sleep laboratory. Although no published data support the use of CPAP among chronic (as opposed to acute) post-cerebrovascular event patients specifically for the reduction of vascular events, there is considerable evidence to support the treatment of sleep apnea in general to improve a variety of patient outcomes [35, 47]. Our findings add to the growing literature that suggests that the effect of CPAP on blood pressure is modest and likely only present in patients with uncontrolled hypertension. The study results provide a rationale for using polysomnography to identify sleep apnea among all patients post-stroke or post-TIA given its high prevalence, and suggest that support for CPAP adherence should persist over time, because many patients in the intervention group in this study continued to adapt to CPAP well beyond the first month of receiving a CPAP machine.
